# Screening for Language Difficulties in Disadvantaged Populations on Entry to Early Years Education: Challenges and Opportunities

**DOI:** 10.3389/fped.2022.833603

**Published:** 2022-04-25

**Authors:** Julie E. Dockrell, Claire L. Forrest, James Law, Sandra Mathers, Jenna Charlton

**Affiliations:** ^1^Department of Psychology and Human Development, Institute of Education, University College London, London, United Kingdom; ^2^School of Education, Communication and Language Sciences, Newcastle University, Newcastle upon Tyne, United Kingdom; ^3^Department of Education, Social Sciences Division, University of Oxford, Oxford, United Kingdom

**Keywords:** language, preschool, social disadvantage, education, English as an additional (second) language, LUI

## Abstract

Children aged 3–4 years (*n* = 876) were recruited from deprived areas in England, and a significant minority of the sample were second language learners. Oral language ability was assessed using child administered standardized measures, and parents reported on children’s language. We adapted the Language Use Inventory [LUI; (1)] to capture carer’s reports of the children’s structural language in the language of instruction and their home language (where appropriate). The final measure included six subscales from the original: use of simple words, requests for help, gaining attention, talking about activities/actions, interactions with others, and building sentences. Children’s language abilities and non-verbal abilities were below norms on all standardized tests administered except non-word repetition. Factor analysis indicated that all the six scales of the adapted parent completed measure loaded on one language factor. The revised total scale score correlated significantly (*p* < 0.0005) with child assessed language measures, specifically expressive vocabulary and grammar. Different patterns across gender, language status and parental education were examined. Sensitivity and specificity of the scale to identify children with the greatest delays were evaluated. These preliminary data indicated that parent-reported information on children’s language skills at 3 years of age has the potential to provide a reliable indicator to inform pedagogy and practice at the start of nursery school. Study limitations are examined and avenues for future development explored.

## Introduction

Children from areas of social disadvantage experience delays in their language development, with significant numbers of children entering nursery classes with limited oral language skills. These difficulties are exacerbated when a child’s home language is not the language of instruction. This causes challenges for early years practitioners in developing the classroom language learning environment and in targeting resources for the children. There is a need for teachers to access tools to profile children’s language skills to inform their practice. Parent completed measures provide a viable means of assessing oracy skills on entry to nursery school. We examined the feasibility and validity of using a modified parental completed language checklist in areas of social disadvantage as an indicator of children’s language skills as they enter nursery classes.

Language is a foundational skill supporting interaction with others, accessing the curriculum, and developing literacy skills. Proficiency in oral language provides children with a vital tool for thinking and learning (2). Early language delays are a significant risk factor for later literacy and learning difficulties and for longer term unemployment (3). Universal community surveillance estimates prevalence rates of language delays as between 3 and 8% of the population at 30 months of age (4), with children from areas of social disadvantage experiencing disproportionate delays relative to their more advantaged peers (5, 6). Social disadvantage can be measured in different ways, but typically refers to the distal factors (e.g., household income, parental occupation status, parental education, neighborhood poverty) that influence development. Language minority children in areas of social disadvantage experience a double disadvantage (7, 8). There is widespread agreement that additional support is needed to address these inequities, but continued disagreement about the ways in which this should be done. The use of child health surveillance programs to capture language delays (9), targeted parental packages (10), and specific language interventions (11) have all been proposed as ways of addressing the impact of children’s language delays [see also (12)]. These targeted approaches have had varying degrees of success but overall point to the importance of ensuring that all children are provided with evidenced informed universal language support when they enter education [see for example (13)]. Children in at risk contexts are likely to need more systematic “targeted” universal interventions in educational contexts [see (9, 14)]. Despite this reported need, limited use of language supporting strategies have been recorded in these settings in areas of social disadvantage [see for example, (15, 16)], and teachers’ knowledge of oral language pedagogy is not always optimal (17). An essential component of supporting young children’s oracy skills is to provide researchers with reliable and time efficient tools to capture language development at school entry, and early years practitioners with ways of profiling the language performance of children who enter their settings. This is of particular concern in areas of social disadvantage where there is an enhanced need for methodologically robust interventions (18). In this study, we examined whether a parental questionnaire, the Language Use Inventory [LUI, (19)]^1^, that we abbreviated and modified with respect to its standard administration, would provide reliable and valid language performance data for children from areas of significant social disadvantage on entry to nursery classes in schools in England, at the age of three. Nursery settings were included in the study if they were within the lowest quintile of deprivation as measured by the United Kingdom (UK) government Indices of Deprivation 2015 (20). We argue that knowledge of children’s language levels as they enter early learning environments provides educational professionals with the evidence to develop enhanced language learning activities, thereby reducing the likelihood of poorer language learning opportunities (21). However, to achieve this objective, education professionals need access to reliable and valid tools which are cost efficient and can be completed in a time efficient manner.^2^

Staff in early years settings in England are often tasked with profiling and monitoring children’s language levels (22). Access to reliable and valid measures of language performance for children between the ages of three and four is, however, limited (23–26). Many of the assessments that do exist are neither designed for teacher use, nor are they appropriate for monitoring progress. These formal assessments typically take a long time to administer, can be complicated to score, and have specifications about the length of time required between assessments due the psychometric properties of the measures. For example, measures considered gold standard by clinicians for children of this age, such as the Clinical Evaluation of Language Basics Preschool [CELF; (27)], take over 40 min to administer and require clinical training for administration and interpretation. It is perhaps not surprising that teachers report a lack of tools to assess children’s language levels and regard this as a challenge for practice (28).

An additional challenge in the evaluation of children’s oral language for teachers and researchers is the diversity of home languages spoken by children. Schools, where a significant minority of pupils do not speak the language of instruction at home, is now a frequent occurrence (29). In England, over 1 in 5 children has English as an additional language (EAL) (30), and in areas of social disadvantage, these figures are significantly higher. As such, many children are faced with learning and being assessed in the official language of education which is often not their home language. While there are ideological and practical debates about the ways of dealing with multilingual classrooms, the classroom reality is that teachers are working in multilingual contexts in the United Kingdom and Europe (31). An understanding of the language learning needs of language minority children should enhance teaching and learning.

Standardized parental questionnaires provide an alternative means of capturing children’s language levels either in English or a child’s home language, but these have been typically designed for infants and toddlers or for older children. For example, the MacArthur communicative development inventories (CDI) scales can be used for children 36 months and younger (32). For older children, checklists have emphasized wider aspects of communication. The Children’s Communication Checklist—2 [CCC-2, (33)] is a brief clinically relevant caregiver questionnaire which assesses pragmatic language impairments in children and adolescents. The CCC-2 has been the focus of much research, predominantly with children with disabilities and those of school age (34, 35). The measure is not appropriate for use with children younger than 4 years. To address this gap, the LUI (19) was developed. It is a parent-completed questionnaire designed to assess children’s pragmatic language between the ages of 18 and 47 months (1). The original measure, designed for use with children whose primary language is English, is an important contribution to tools available and correlates with later outcomes at school entry on standardized language measures (e.g., CELF-P2; CCC-2), demonstrating high levels of specificity (93%) and Negative Predictive Value (92%) (36). The LUI has been used in a range of contexts and translated into several languages, allowing for use in different country contexts (37–39).

The current study focused on children entering preschool settings. As such, we abbreviated the original scale to focus on structural language (vocabulary and grammar). Both vocabulary and grammar are key dimensions of children’s oral language as they enter school in England (40), and profiling performance on both dimensions has the potential to inform classroom pedagogy for at risk groups from areas of significant social disadvantage. In addition, given the multilingual context of English schools where, for a significant minority of children, their home language is not the language of instruction, we modified the original administration and scoring (19) to allow parents to complete the form with reference to both English and their home language (see “Materials and Methods” section for details).

To capture language profiles at this point in development for young children at risk of language delay, it is important to establish which language skills should be measured to provide staff with indicative levels of performance and need. It is well established that children who enter school with small vocabularies and limited grammatical skills (41, 42) experience difficulties in accessing the curriculum and have lower levels of attainments in school (43). Significant numbers of children from lower-socioeconomic status (SES) backgrounds have large gaps in their vocabulary (5), negatively impacting learning (44). Growth in oral language during early childhood reflects a continuous development of lexical representations (vocabulary) and the development of an implicit understanding of the rules of grammar. Both these skills have been described as core language components (40, 45). These skills underpin later development of narrative language which can be reliably captured in typically developing children above 4 years of age (40, 45).

There is also increasing evidence that capturing expressive language is crucial. Not only is the development of the ability to use high quality talk in the classroom increasingly recognized as a key component of children’s education (46), children’s expressive language predicts the quality of language provision they receive (47). Teachers also use spoken language to assess learning (48), and their perceptions of expressive language ability have been significantly correlated with their perceptions of a child’s overall development (49). As such, reliable measures of expressive language can provide teachers with data to make evidence based decisions. Finally, agreement between parent report and direct assessment is stronger for language production than for comprehension (50). These studies suggest that, to profile oral language skills for pedagogical purposes, both expressive vocabulary and grammar should be sampled in young children whose language is likely to be delayed.

There are several advantages in using parental report as children first enter nursery school. Parents have had the opportunity to observe and interact with their children across various situations (50), opportunities that nursery staff will not yet have experienced. Moreover, children take time to adapt to nursery settings and their respective routines and, as such, may not use language as competently as they can in familiar settings with familiar people. It is also the case that many of the children in these at-risk settings come from families where their home language is not the language of instruction, English, in the context of this study. As such, parents will have a broader understanding of the diverse ways in which children can use language. Parental reporting, at this point in development, has already been systematically embedded within routine pediatric developmental screening, often to identify at risk children (51, 52). Involving parents in providing information about children’s language abilities also brings benefits in terms of enhancing parent-school relationships (since parental knowledge is being valued and requested by the school) and parent involvement in children’s schooling, both of which have been shown to predict children’s academic and social outcomes (53). The potential for using similar approaches in early years educational settings to develop language learning pedagogy requires further exploration.

## The Current Study

We reasoned that a modified version of the LUI (1), focusing on children aged 3–4 years of age as they take their first steps in universal education, had the potential to provide teachers with a profile of children’s oral language strengths and needs. Given our focus on children from areas of significant social disadvantage, the LUI items were modified in three ways. Firstly, to shorten completion time for parents, the four subscales which are not included in the original LUI Total Score and its norms were omitted (Subscales A and B on gestures, and Subscales E and L with open-ended questions about the child’s interests in play and talk). Secondly, we prioritized subscales with items related to language use with developmentally later-emerging expressive vocabulary and grammar, given their importance for later academic achievement [see for example (54, 55)] and that evidence that expressive narrative skills in 3-year-olds from disadvantaged settings may be too limited to provide a useful focus of assessment (56). Thirdly, we sought to prioritize subscales with potential to provide feedback to teachers about their children’s language levels and ultimately inform targeted interventions (57). Six of the 10 scored LUI subscales met our criteria, with three focusing primarily on vocabulary (*C: Types of words your child uses, F: How your child uses words to get you to notice something, I: Your child’s use of words in activities with others)* and three focusing on more extended language use and grammar (*D: Your child’s requests for help, H: Your child’s questions and comments about themselves or other people, N: How your child is building longer sentences and stories*). These six subscales all had high factor loadings on the original scale of 0.83–0.95 [see (19)]. We will refer to our abbreviated version of the LUI as the LUI-6 henceforth. Scales are reported as described in the original LUI.

Given the focus on educational settings, we also modified the original administration and scoring (19). We adapted the LUI from one response column to two response columns. This allowed parents to separately record children’s abilities in English and their home language. The LUI-6 English is used to refer to parents’ reported use of English. When we are referencing reported use of English on the LUI-6, we refer to it as LUI-6 English. Language status was operationalized in the current context as either monolingual English or different home language. We first examined responses for English to establish whether the LUI-6 was reliable and valid and reflected the same factor structure as the original. Secondly, to examine validity, we compared responses for the LUI-6 with scores on a standardized child administered measures of oral language. Thirdly, we examined whether performance on the LUI-6 provided reliable data to identify the children who performed below 1.5 SDs from the mean on standardized measures of oral language. Finally, we explored the data provided by a sub-sample of parents who recorded their child’s language in both English and a home language to establish whether there were differences between reports for English and home language. Given the current understanding of oral language development at age three, we predicted that responses for the six subscales from the LUI-6 would load on a single factor as in the original research (1). We also anticipated that, as with the original LUI, the LUI-6 would correlate significantly with child administered standardized measures of the children’s expressive and receptive oral language. We anticipated that the focus on expressive language in the LUI-6 would result in high levels of sensitivity and specificity in identifying children who were struggling most with English at this point in development. Assessment of home language skills raises many challenges and, while lags in the language of instruction are often evident, there have been fewer attempts to capture differences in performance between the language of instruction and home language in disadvantaged populations.

## Materials and Methods

### Ethics

Ethical approval was granted by Institute of Education (IOE) Research Ethics Committee (IOE REC 1118: Empowering Staff to Enhance Oral Language in the Early Years).

### Participants

The current sample formed part of a larger intervention study investigating the language learning needs of children from disadvantaged areas in England (58). A power calculation using G*Power (Cohen’s *d* > 0.20, *p* < 0.05) determined that a target sample of 600 participants was needed to observe an intervention effect. Schools that were in the lowest quintile for deprivation in the United Kingdom and contained a nursery class were recruited in Greater London and Teesside (in the Northeast of England). Settings were recruited via flyers advertising the study, presentations given to school partnerships, and a meeting of Early Years leaders from local primary schools. The project was also publicized through local charities, language leads, and social media. Deprivation information was generated from nursery postcodes using the Income Deprivation Affecting Children Index (IDACI), one of the subscales of the UK government’s English Indices of Deprivation 2015 (20). The IDACI measures the proportion of all children aged 0–15 years living in income-deprived families and ranks small geographical areas in England from 1 (most deprived) to 32,844 (least deprived). IDACI Ranks were generated from each nursery’s postcode. These scores were converted to a z-score using the normsinv function on excel and dividing the IDACI Rank by 32,844, as recommended by Bishop (59). Thirty-seven nurseries in Greater London expressed interest, of which 28 met inclusion criteria for deprivation. Five nurseries did not respond to invitations to participate, and two nurseries declined to participate. Thirty nurseries in Teesside which met inclusion were approached. Seven nurseries did not respond to invitations and three nurseries declined to participate. In total, 41 nurseries were recruited into the study, but two nurseries, one from each area, withdrew, resulting in a final sample of 39 nurseries (London *n* = 20; Teesside *n* = 19).

A total of 876 children were recruited into the sample, 29 of whom were not eligible because either: their parent or teacher reported special educational needs (SEN) (*n* = 14); children no longer attended the nursery (*n* = 9); children were wrongly recruited (too young or in a different class) (*n* = 4); or teacher reported no English (*n* = 2). A further 25 children did not complete the assessments due to absences (*n* = 9) or refusals (parent withdrew consent/incomplete consent *n* = 3; child declined to participate *n* = 13).

Demographic details were collected from the parents, and parents were also asked whether they had any concerns about their child’s language development. Demographic details are presented in Table 1. As the table shows, 42.4% of the children spoke English as an additional language (EAL, *n* = 331). There were 47 different languages spoken in the sample (*n* = 19 European, *n* = 14 African, *n* = 6 South Asian, *n* = 5 East Asian, *n* = 2 Middle Eastern and *n* = 1 Caribbean). More children were from bilingual homes in the London sample [χ^2^(1, 823) = 276.97, *p* < 0.001], and more children attended nursery part-time in Teesside [χ^2^(1, 801) = 57.44, *p* < 0.001]. No other differences were statistically significant.

**TABLE 1:** Demographics of sample.

	London (*n* = 438)	Teesside (*n* = 384)	Total sample (*N* = 822)
Child characteristics			
Mean Age in months (SD)	43.32 (3.90)	43.82 (3.95)	43.56 (3.93)
Female (%)	49.5	54.4	51.8
First born (%)	33.3	31.9	32.6
Reported language concern (%)	20.1	12.6	16.5
Home language (%)			
Monolingual	30.0	87.7	57.6
English as an additional Language	70.0	12.3	42.4
Parent/carer characteristics			
Relationship to child (%)			
Parent	98.5	95.9	97.4
Other	1.4	4.1	2.8
Highest level of education (%)			
Primary school	8.2	8.5	8.3
GCSE level	19.9	24.8	22.2
Above	71.9	66.7	69.5
Frequency of book reading (%)			
Every day	38.6	41.4	39.9
At least once a week	60.1	57.3	58.8
Never	1.2	1.4	1.3

### Measures

Children’s language abilities were measured in two ways: first, by direct assessment of the child’s language skills in the nursery setting and, secondly, by asking parents to complete the LUI-6. Details of the measures are discussed below.

### Child Assessment

#### Expressive Language

The Naming Vocabulary subtest of the British Ability Scales 3rd Edition [BAS-3; (60)] assesses children’s knowledge of nouns. Children are required to name colored pictures of objects shown one at a time. Successful performance depends on expressive language ability, picture recognition skills, and long-term memory skills. Naming vocabulary has a test-retest reliability of 0.92, and internal reliability coefficients range from 0.70 to 0.92 between the ages of 3 and 4 years.

The sentence repetition component of the Grammar and Phonology Screening test [GAPS; (61)] is another measure of expressive language, designed to highlight significant markers of language impairment and reading difficulties. Children repeat 11 sentences that target subject-verb agreement, tense marking (past, future), phrasal embedding, dative construction, object question formation, reversible passive construction, and anaphoric and pronomial reference. All words have an early age of acquisition, simple phonological structure, and knowledge of the words is reported to be independent of socioeconomic and cultural bias. The sentence repetition component has a reliability of α = 0.86 and is significantly correlated with the CELF Sentence Structure (*r* = 0.52). Sentence repetition tasks are very sensitive markers for identifying children with developmental language disorder (62).

#### Receptive Language

The Verbal Comprehension subtest from the BAS-3 (60) provides a measure of receptive language by assessing children’s understanding of oral instructions involving basic language concepts. Children respond by either pointing to a picture, handing objects to the researcher, or placing objects in different positions according to the instructions. Questions assess children’s understanding of object names, commands (e.g., “Put the horse in the box”), the functions of objects (e.g., “Give me the one we draw with”), prepositions (e.g., “Put the car under the bridge”), and complex instructions (e.g., “Give me all the red shapes except the square.”). Verbal Comprehension has a test-retest reliability of *r* = 0.78 and internal reliability coefficients range from 0.85 to 0.91 between the ages of 3 and 4 years.

#### Phonological Skills

The non-word repetition subtest from the GAPS (61) requires children to repeat eight non-words which vary in complexity (e.g., *dremp, bademper, difimp*, etc.). It has reliability of α = 0.73 and construct validity of *r* = 0.58 with the Children’s Test of Non-word Repetition [CNRep; (63)].

#### Narrative Skills

The Bus Story test (64) is a measure of story retell abilities while also drawing on verbal comprehension. The researcher reads aloud a story about a “naughty bus,” and children follow along looking at the pictures. Children are then asked to tell the researcher what happened in the story using the pictures as guides. The Bus Story was administered on a laptop, and children’s responses were recorded with the in-built microphone and by hand. Research assistants transcribed the recordings and then scored the responses for information (the amount of information the child conveys when telling the story) using the published coding system. Participants receive a score of 2, 1, or 0 for each item, depending on the amount of detail that has been reported. There are a total of 54 points that can be obtained. Information has a test-retest reliability of 0.79 and construct validity of 0.98. Ten randomly selected transcripts were coded by the lead researcher (CF) and two research assistants using the scoring guidelines. Any discrepancies in scoring were discussed, along with the rationale for scores, until agreement was reached. Any decisions were documented, and a further ten random transcripts were coded using these guidelines. Again, discrepancies were discussed until agreement was reached. The researchers then coded 20% of the complete Bus Story assessments (*n* = 149 transcripts) which were randomly selected. Inter-rater reliability on these 149 transcripts was calculated using intra-class correlations (ICC). A mean-rating (*k* = 3), absolute-agreement, two-way mixed effects model was used to calculate ICC in SPSS v25.0 (65). This model treats the rater as a fixed effect and the Information Score as a random effect because we are only interested in the reliability between the three raters who coded the same 149 transcripts (66). Inter-rater reliability coefficients were excellent [ICC = 0.9 (95% CI = 0.97–0.99)] (67).

#### Non-verbal Abilities

The non-verbal reasoning ability cluster from the BAS-3 (60) consists of the Matrices scale and the Picture Similarities subtest. Both subtests were administered on a laptop. The Matrices scale assesses children’s perception and application of relationships among pictures or abstract figures. Children are shown a 4 × 4 matrix, three of which are colored objects and one is blank. Below the matrix are four options from which to choose the missing object that fits the pattern. There are minimal oral instructions, and children respond by pointing. Internal reliability for the Matrices subtest ranges from 0.68 to 0.83 between the ages of 3 and 4 years and has a test-retest reliability of *r* = 0.64. The Picture Similarities subtest requires children to match a picture to one of the four pictures displayed. This task requires minimal oral instruction, can de demonstrated by gesture, and children do not need to respond verbally. The Picture Similarities subtest has an internal reliability ranging from 0.68 to 0.83 between the ages of 3 and 4 years and a test-retest reliability of *r* = 0.64. Overall, the non-verbal reasoning ability cluster has a test-retest reliability of *r* = 0.77 and an internal validity of *r* = 0.46.

### Parental Report of Language Ability

The LUI (19) is a standardized parent-report measure of language use in daily life for children aged 18–47 months. Parents report on children’s current language and communication skills using a nominal scale (Yes/No) or ordinal scale (Never/Rarely/Sometimes/Often). Parents were instructed to complete the questionnaire in 1 day and were encouraged to ask others for help if needed (e.g., partner, child’s grandparent, child’s nursery teacher). The LUI was designed to assess young children’s spoken pragmatic language use with a focus on the functions of language influenced by children’s developing cognitive abilities and increasing social interactions during this age (1). As described above, six of the original 10 scored LUI were used, namely, *C: Types of words your child uses; D: Your child’s requests for help; F: How your child uses words to get you to notice something; H. Your child’s questions and comments about themselves or other people*; *I: Your child’s use of words in activities with others;* and *N*: *How your child is building longer sentences and stories*.

The items chosen assesses later appearing and more sophisticated uses of language that involve greater vocabulary, grammatical, and conversational skills (68). The original version of the LUI has good internal reliability, ranging from 0.83 to 0.98 (1, 19). Five minor word changes were made to example phrases provided to reflect a British sample (e.g., mommy to mummy; airplane to airplane).

Finally, the format of administration was modified. The original instructions allow parents to reply regardless of language used by the child. This was modified so that, in this study, parents could indicate, for each item, in their reply whether the child was able to produce the target item in English or their home language (if appropriate). The current data report on the nominal items (omitting 9 ordinal items) for which parents replied with respect to their child’s use in English (the LUI-6 English) and, where appropriate, the home language, with a total of 111 English items scored 1 (Yes) or 0 (No).

In the current study the standardization from the LUI does not apply given the use of selected subscales from LUI such that the full range of abilities were not sampled, and parents were not given the standard instructions as per the LUI Manual.

### Procedure

Nursery settings were recruited into the intervention study spring/summer 2019. Informed consent and background questionnaires were delivered to parents by nursery staff. A team of five research assistants and one research associate conducted the 1:1 child assessment sessions during the autumn term. All assessors completed comprehensive training, and their performance was assessed at the end of the training. Each researcher spent approximately 1 week at each setting administering the assessments in two 20-min sessions. Children received a sticker after each session as a thank you for their participation. Parents received an envelope containing the LUI-6 after their child had been assessed with instructions to complete and return to the nursery within a week. Envelopes and questionnaires were labeled with a unique ID for each child to maintain anonymity. Nursery staff were encouraged to support parents in the completion of the questionnaires when necessary.

### Statistical Analysis

Data were analyzed using SPSS v25. Chi-square analysis was used to compare group differences between those who did and did not return the LUI-6, examining background demographics and child performance on direct assessments of language. Factor analysis of the LUI-6 was conducted using principal component analysis with varimax rotation to examine the factor loading for the LUI-6. Subsequently, group differences on the LUI-6 factor score were examined using an ANOVA with gender, language status (monolingual or bi/multilingual), and parent report of language concern entered as fixed factors. Concurrent validity of the LUI-6 factor score was analyzed using zero-order correlations to explore the relationship between the LUI-6 factor score and scores from the directly assessed language measures. The effect of directly assessed language ability on the LUI-6 factor was assessed using stepwise regression by entering language status first. A receiver operating characteristic (ROC) analysis was conducted to measure the sensitivity and specificity of the LUI-6 in identifying children with the lowest language ability (more than 1.5 SDs below the mean). Finally, differences between English language and home language use as reported by the LUI-6 were compared using *t*-tests, and principal component analysis was used to explore the factor loadings of the LUI-6 when home language was reported.

## Results

The results are presented in five sections. In the first section, we examine differences between the parents who returned a completed LUI-6 for their children (for English, home language or both) and those who did not. Significant numbers of parents did not complete or return the questionnaire, and we reasoned that these data inform interpretation of the subsequent results. Section 2 provides data on the pattern of responses across the six subscales used and examines the factor structure of the LUI-6 using principal component analysis to establish whether the six subscales formed unitary construct (as with the original LUI) or several constructs. In Section 3, we examine the concurrent validity of the LUI-6 by exploring parental responses with direct child assessment on the language measures collected from the children. Section 4 explores the sensitivity and specificity of the LUI-6 in identifying children with the most delayed language, and, finally, Section 5 explores responses related to the children’s home language, where reported.

### Section 1: Parental Completion of the Language Use Inventory-6

Of the final 876 participants in the sample, a total of 338 LUI-6 forms were returned (38.6% of the total sample). Of these 338 completed forms, 225 were completed with respect to the English only (66.6%), and a further 110 (32.5%) were completed with reference to both spoken English and the child’s *spoken* home language. Only three forms (1%) were completed with respect to solely the child’s use of their home language, and these are not considered further in the analyses.

The LUI-6 was more likely to be completed by parents of monolingual English children [χ^2^(1, *N* = 794) = 9.80, *p* = 0.002] who were from less deprived catchment areas [*t*(837.255) = 3.34, *p* = 0.001; LUI-6 completed *M* = −1.60, *SD* = 0.469; not completed *M* = −1.71 *SD* = 0.61], and who had children who scored higher on the BAS-3 oral language measure [*t*(794) = 2.68, *p* = 0.008: LUI-6 completed *M* = 87.68, *SD* = 14.97; not completed *M* = 84.81, *SD* = 14.65]. Where significant differences were evident, either the association was weak (monolingual English Cramer’s *V* = 0.105) or the effect sizes were small (Indices of deprivation Cohen’s *d* = 20; oral language measure Cohen’s *d* = 0.19). There were no differences between the educational levels of parents who returned the form and those who did not [*t*(642.313) = −4.77, *p* = *ns*: LUI-6 completed *M* = 3.15, *SD* = 1.11; not completed *M* = 3.26, *SD* = 1.03), child age *t*(865) = 1.162, *p* = *ns*: LUI-6 completed *M* = 43.66, *SD* = 3.83; not completed *M* = 43.34, *SD* = 3.99)], or parental concern about the child’s language [χ^2^(1, *N* = 808) = 0.005, *p* = *ns*]. In sum, there was evidence of some bias in response rates where forms were less likely to be completed by the parents of bilingual/multilingual children, children with lower language levels, and those from the most deprived settings.

### Section 2: Performance on Language Use Inventory-6 Subscales

We first consider responses related to children’s use of English (either when only completed in English or when completed in addition to home language), reflecting the language of instruction and the original development of the LUI scale. In the final section where data are reported (see Table 7), we examine responses for home language (among those who also reported use of English) and compare these with responses for English.

**FIGURE 1:**
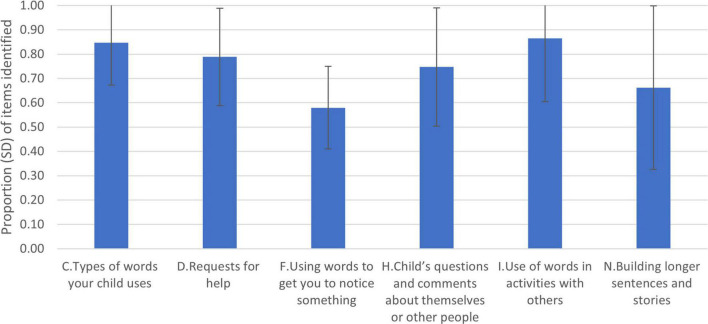
Mean proportion of items (SD) recorded correct for use of English for each of the LUI subscales employed.

Table 1 reports the responses to the different six subscales used from the original LUI. Figure 1 shows the proportion correct for each scale to allow comparison across the six subscales. To test the overall internal consistency of all the subscales together, we first computed Cronbach’s alpha. As with the full LUI, Cronbach’s alpha was high for the selected subscales, 0.979. Children’s mean scores (and SD) for each subscale are provided in Table 2. Apart from subscale N, *How your child is building longer sentences and stories*, the subscales were negatively skewed, indicating that children’s reported performance was high. This was most marked for subscale C, *Types of words your child uses* (skewness -3.224, *SE* = 0.133).

**TABLE 2:** Children’s raw scores on the six LUI subscales based on parental reported use of English (sole or in addition to home language) (*N* = 335).

LUI Subscale	Number of items	Mean	*SD*
C: Types of words your child uses	21	17.78	3.67
D: Your child’s requests for help	7	5.52	1.4
F: How your child uses words to get you to notice something	6	3.48	1.02
H: Your child’s questions and comments about themselves or other people	36	26.9	8.74
I: Your child’s use of words in activities with others	14	12.1	3.64
N: How your child is building longer sentences and stories	36	23.82	12.12

To explore relative performance across the subscales, proportion scores for each subscale were calculated. These are shown in Figure 1. Both subscale F *‘How your child uses words to get you to notice something’* and subscale N “*How your child is building longer sentences and stories*’ were subscales where the lowest proportion of items were reported by the parents for the children in the study. A non-parametric Friedman test of differences on proportion scores was conducted, and there was a significant difference between the subscales [χ^2^(5) = 822.27, *p* < 0.0005]. Dunn-Bonferroni *post hoc* tests were carried out, and there were significant differences between the subscale F *‘How your child uses words to get you to notice something’* and all other subscales (all *ps* < 0.0005). Performance on subscale N “*How your child is building longer sentences and stories*” was significantly lower than subscale C *“Types of words your child uses”* (*p* < 0.0005) and subscale I “*Your child’s use of words in activities with others”* (*p* < 0.0005). The data reflect developmental trends in performance found in the original studies (1, 19).

Following O’Neill (19), we examined the factor structure of the revised scale. Principal Component Analysis was conducted with varimax rotation. As Table 3 shows, all subscales loaded on a single factor accounting for 79.6% of the variance, with loadings for each scale on the factor all above 0.80. As one factor was identified, the regression-based factor score is used in subsequent analyses to identify ranking on the latent variable and allow follow-up analyses. Factor scores are standard scores with a Mean = 0 and Variance = squared multiple correlation between items and factor. This procedure maximizes validity of estimates. This latent variable is referred to as LUI-6 factor.

**TABLE 3:** LUI-6 subscales principal component analysis, rotated factor loadings based on parental reported use of English (sole or in addition to home language) (*N* = 335).

LUI subscales	Factor loadings
C: Types of words your child uses	0.867
D: Your child’s requests for help	0.887
F: How your child uses words to get you to notice something	0.901
H: Your child’s questions and comments about themselves or other people	0.943
I: Your child’s use of words in activities with others	0.942
N: How your child is building longer sentences and stories	0.806

We examined differences on the LUI-6 factor score by gender, language status (monolingual or bi/multilingual), and whether parents had reported concerns about child’s language. An analysis of variance yielded a main effect for monolingual English [*F*(1,301) = 12.16, *p* = 0.001, η*_*p*_*^2^ = 0.04 (monolingual English speakers LUI-6 English factor score *M* = 0.202, *SD* = 0.856; bilingual/multilingual LUI-6 English factor score *M* = −0.308, *SD* = 0.1.109)] and reported language concern *[F*(1,301) = 8.02, *p* = 0.003, η*_*p*_*^2^ = 0.03 (no language concern LUI-6 English factor score (*M* = 0.101, *SD* = 0.938); language concern LUI-6 English factor score (*M* = −0.383, *SD* = 0.1.101)], but there was no effect for gender [*F* (1,301) = 0.031, *p* = *ns* (female LUI-6 English factor score *M* = 0.114, *SD* = 0.91); Male LUI-6 English factor score (*M* = −0.072, *SD* = 0.1.040)]. In sum, differences on the LUI-6 factor score were evident for language status and concern about children’s language skills, but effect sizes were small, accounting for only 4% of the variance for language status and 3% of the variance for parental concern. There were no significant interaction effects.

### Section 3: Concurrent Validity of Language Use Inventory-6 Factor Score Based on Parental Reported Use of English (*N* = 335)

Given the six subscales from the original LUI had been selected to reflect vocabulary and grammatical aspects of oral language, we examined the LUI-6 factor score based on parental reported use of English (sole or in addition to home language) in relation to the scores children achieved on the direct assessments of oral language captured in the study.

As Table 4 shows, there were significant correlations between the LUI-6 English factor score and all language measures except the Bus Story sentence length. As predicted, correlations with standardized measures of expressive vocabulary and grammar were large, demonstrating high levels of concurrent validity between parent reported English use on the LUI-6, and these standardized directly administered child measures. Relationships between the LUI-6 and non-word repetition and narrative skills, while significant, were lower. Correlations between naming vocabulary and sentence repetition and the LUI-6 factor score were statistically significantly higher than those between the LUI-6 factor score and Bus Story sentence length (*Z* = 10.732, *p* < 0.001), non-word repetition (*Z* = 8.331, *p* < 0.001), Bus Story information (*Z* = 6.311, *p* < 0.001), and Verbal comprehension (*Z* = 2.337, *p* = 0.01), providing further evidence that the LUI-6 is capturing lexical and grammatical expressive oral language in this population.

**TABLE 4:** Zero order correlations between LUI-6 factor score and directly assessed language skills (*n* = 312).

	LUI-6 factor score	GAPS: sentence repetition	GAPS: non-word repetition	BAS: verbal comprehension	BAS: Naming vocabulary	Bus Story information
GAPS: sentence repetition	0.411**					
GAPS: Non-word repetition	0.206**	0.585**				
BAS: Verbal comprehension	0.533**	0.647**	0.431**			
BAS: Naming vocabulary	0.640**	0.543**	0.273**	0.723**		
Bus Story: Information	0.322**	0.555**	0.315**	0.527**	0.502**	
Bus Story: Sentence length*^a^*	0.062	0.368**	0.292**	0.215*	0.300**	0.531**

^a^Only 127 children produced Bus Story narratives that were sufficiently long to complete a sentence length score. *Correlation is significant at the 0.01 level. **Correlation is significant at the 001 level.

Using regression analyses, we examined whether direct assessments of children’s language contributed significantly to the LUI-6 English factor score once language status (monolingual or bi/multilingual) was accounted for. Language status was entered first, followed by the standardized measures of oral language and parental concern about children’s language levels. This allows a test of whether performance on the standardized language measures accounted for performance on the LUI-6 score once language status was considered. We hypothesized that both language status and expressive language would account for the most variance in the LUI-6 factor score. A significant model was found when only language status was used in the model [*F*(1, 334) = 25.16, *p* < 0.0001, *R*^2^ = 0.07, *R*^2^*_*Adjusted*_* = 0.067], but the model only accounted for a small proportion of the variance. Inclusion of the directly assessed language measures in the second step significantly improved the model, resulting in a significant *R*^2^ *change* = 0.266 and a model that accounted for over 30% of the variance in the LUI-6 score [*F*(6, 334) = 27.66, *p < 0.0001, R*^2^ = 0.346, *R*^2^*_*Adjusted*_* = 0.324]. As Table 5 shows the final model language status is no longer significant and responses to the LUI-6 are explained by children’s performance on direct assessments of naming vocabulary and verbal comprehension.

**TABLE 5:** Final regression model examining predictors of performance on the LUI-6 factor based on parental reported use of English (sole or in addition to home language) (*N* = 335).

	*B*	Std error	Beta	*t*	Sig
Language status	–4.645	2.834	–0.801	–1.639	*ns*
GAPS sentence repetition	0.618	0.541	0.78	1.143	*ns*
GAPS non-word repetition	0.036	0.579	0.003	0.061	*ns*.
Verbal comprehension	0.281	0.085	0.219	3.319	0.001
Naming vocabulary	0.371	0.066	0.340	5.600	<0.0005
Bus story information	–0.174	0.400	–0.024	–0.436	*ns*

*ns, non significant.*

### Section 4: Capturing the Children With the Greatest Levels of Language Learning Need

To identify whether scores on the LUI-6 English factor score accurately identified children who had the poorest levels of language, we computed a ROC analysis. ROC analysis provides data on which variables offer the best discriminatory power in this study to identify children with the lowest levels of language. If the area under the curve is 1, this would illustrate perfect discrimination with 0.5 being chance.

We used standard scores on the BAS-3 verbal ability measure as our benchmark level of language performance. Following data which suggest that significant children struggling with oral language can be captured when scores are more than 1.5 SDs below expectation, children who performed more than 1.5 SDs from the mean were classified as the poorest performers (33.4%, *n* = 108). To capture the utility of the LUI-6 English factor score, we compared performance on the parent-completed LUI-6 questionnaire with two direct assessments of children’s expressive language not used to identify poorest performance in oracy (GAPS sentence repetition and the Bus Story information score). As Table 6 shows, all measures demonstrated good sensitivity and specificity in identifying the children with the poorest levels of language and, importantly, the LUI-6 English factor score performed at a similar level to direct assessments of the children’s language levels.

**TABLE 6:** ROC analysis for LUI-6 English factor score and direct language assessments in identifying children with the greatest levels of language learning need: area under curve and 95% CI.

	Area under the curve	Std error	Significance	Lower bound	Upper bound
LUI-6 English factor score	0.780	0.033	<0.0005	0.714	0.845
GAPS sentence repetition	0.806	0.027	<0.0005	0.752	0.859
Bus story information	0.795	0.028	<0.0005	0.740	0.849

**TABLE 7:** Comparison between scores for LUI-6 subscales when reported for English language use and home language use for the same child.

LUI subscale	LUI-6 reported Home Language *M (SD)*	LUI-6 reported English M (*SD*)	*t* all sig < 0.0005	Cohens *d*	95% CI
C: Types of words your child uses	10.10 (7.48)	15.58 (5.63)	5.529	0.54	0.33–0.74
D: Your child’s requests for help	2.95 (2.13)	4.79 (1.68)	4.923	0.48	0.28–0.68
F; Your child’s use of words to get you to notice something	1.57 (1.28)	2.98 (1.37)	5.918	0.59	0.37–,78
H: Your child’s questions and comments about themselves and other people	12.85 (12.91)	22.26 (11.52)	4.818	0.47	0.27–0.64
I: Your child’s use of words in activities with others	5.72 (5.89)	10.21 (5.00)	5.033	0.49	0.29–69
N: How your child is building longer sentences and stories	8.84 (12.12)	17.20 (12.62)	4.378	0.43	0.23–0.63

### Section 5: Language Use Inventory-6 Subscale Scores Reported for Home Language (in Addition to English)

There is a dearth of knowledge about children’s performance on home languages in multilingual settings. As such, we explored parental responses for children’s home language use (which, among this group, was always in addition to reporting English use). A significant minority of our sample completed the LUI-6 with a reference point of the child’s home language (*n* = 110) in addition to English language performance. As such, we examined the LUI-6 home language scores. The internal consistency for the LUI-6 Home Language was good with Cronbach’s alpha = 0.865. Our first step was to compare reported performance in English and the child’s home language on the LUI-6 subscales. As Table 7 shows, across all six subscales, parents reported greater levels of oracy skills in English than in the home language. For all comparisons, effect sizes were large.

As with the LUI-6 English, we examined the factor structure of the LUI-6 Home Language. Principal Component Analysis was conducted with varimax rotation. As Table 8 shows, as for English, all six subscales loaded on one LUI-6 Home Language factor which accounted for 81.1% of the variance with all subscales loading above 0.80 on the factor.

**TABLE 8:** LUI-6 home language principal component analysis, rotated factor loadings.

LUI subscale	Factor loadings
C: Types of words your child uses	0.868
D: Your child’s requests for help	0.901
F; Your child’s use of words to get you to notice something	0.920
H: Your child’s questions and comments about themselves and other people	0.947
I: Your child’s use of words in activities with others	0.925
N: How your child is building longer sentences and stories	0.840

As with performance on the LUI-6 English, we examined whether there were differences by the child’s gender and parents report of concern about the children’s language development. As with the score LUI-6 score in English, there was no statistically significant difference by gender [*t*(103) = 0.165, *ns*], but scores for children where a concern about oral language was reported (*n* = 19) were significantly lower [*t*(32.07) = 2.36, *p* = 0.024].

## Discussion

Oral language skills are foundational for learning and attainment in school, and it is well established that, on average, children coming from disadvantaged backgrounds experience challenges with both vocabulary and grammar (3, 5, 69, 70). Despite the evidence, there has been little attention paid to enhancing teachers’ knowledge about how to capture children’s language learning skills in the early years. Such evidence could inform classroom pedagogy and practice in the early years of schooling (71). To address this gap in the literature, we used the LUI (19), abbreviated to six subscales, for use with children from areas of social disadvantage as they entered nursery classes in England. As predicted, the LUI-6 subscales all loaded on a single language factor. In addition, the LUI-6 English provided reliable and valid data, correlating with child administered measures of oral language. As anticipated, the LUI-6 resulted in high levels of sensitivity and specificity in identifying children who were struggling most with English at this point in development. By contrast, our exploration of the parents’ responses in children’s home language did not provide evidence of differential performance in the child’s home language. These results are discussed in detail below.

The subscales of the original LUI (19) were ordered to capture early to later emerging social pragmatic functions of language use, as validated in studies with the LUI and its standardization (1, 19, 39). Our data demonstrated similar developmental trends across the six subscales when the focus was primarily on expressive vocabulary and grammar as these are foundational for later language use. In a sample of children from areas of significant social disadvantage, the data showed that language performance can be captured by parental data. There were statistically significant differences between the scales selected from Part 2 of the original LUI (Your child’s communication with words) and those selected from Part 3 (Your child’s longer sentences). The majority of the children in our sample were using words for earlier appearing functions (e.g., Subscale C: *Types of words your child uses*), whereas performance was lower for later-appearing functions more likely to involve longer sentence constructions (e.g., Subscale N: *How your child is building longer sentences and stories*) and *use of words to get you to notice something* (Subscale F) were significantly lower.

Data are preliminary but, if validated in wider settings, there are potential pedagogical implications. Children who rely on short or single word utterances at the age of three are at risk. The data from the Bus Story indicated that the children had difficulties in producing narratives and most of the sample (both monolingual and those with English as an additional language) could not produce five sentences to compute an average sentence length score on the standardized measure. Research has shown that difficulties in producing extended discourse and using language to engage others limits children’s ability to communicate with others in social settings and their ability to actively engage with classroom activities (72, 73). Discourse skills, critical for later achievement, are built on vocabulary and grammar but also from children’s ability to engage in conversational turns. There is a need to move beyond a focus on vocabulary and reducing the word gap in interventions (41) to provide opportunities for extended discourse, to enhance the classroom language learning environment (74, 75), and to develop strategies and resources for their children at entry to nursery (76).

The LUI-6 English was statistically significantly associated with robust child administered measures of oral language. This speaks to the validity of the LUI-6 English, but also has important practical implication. Standardized language tests that need to be administered are time consuming and, typically, teachers do not have access to these standardized measures. The regression analysis indicated performance on the LUI-6 English was predicted by children’s performance on the standardized tests and parental concern about language development, and not on whether they were monolingual English. As such, it was performance as captured by the LUI-6 English that identified children’s language performance, not whether they spoke English as an additional language. These results could provide teachers with data to target their universal support (12). In addition, ROC analyses indicated that the LUI-6 English identified the children who were struggling most with oral language in English. Combined, these data point to the utility of the scale in capturing performance in English on entry to nursery school, providing teachers with evidence to embed more targeted support for oral language with these children and monitor progress systematically (57). The use of a parent-completed measure has the potential to supplement teachers’ own assessments of children’s language and, arguably, to provide a more nuanced picture since parents can provide information about the diverse ways in which their child uses language across multiple contexts and in multiple languages (where relevant). Fostering acceleration requires high-quality, intentional language modeling, and instruction within preschool classrooms (77). Knowing which oral language dimensions to target and which children to provide additional support is the basis for evidence-based practice (78).

We found no differences in reported performance on the LUI-6 for gender either reported for English or home language. This appears to contrast with other studies documenting the early (age 2) linguistic superiority of girls (79) in children who are disadvantaged (80) and the heightened risk of boys for developmental language difficulties (81). Data vary in how long this difference between genders is maintained, with the difference being less evident from 28 months when boys seem to have an increased language learning trajectory and catch up to girls (82). The mean age of our participants was 3;6, and the data suggest that in this population, at this age, gender differences were not evident.

The data also indicated that parental concern, while accounting for a small proportion of variance, was also a predictor of children’s language status in both English and home language. These data further support the importance of schools engaging with parents to explore their views about their children’s language development. While the checklist format of the LUI-6 reduced parents’ need to provide written text and the readability of the questionnaire was of primary school level, some parents may have found understanding the questions and making judgments challenging, thereby resulting in a reduced completion rate. While questionnaires to parents often result in low completion rates, the significant number of unreturned forms (61.4%) raises challenges for use in disadvantaged populations. In particular, this group was more likely to consist of parents who reported speaking a language other than English, who came from more deprived areas, and who had concerns about their child’s language. These findings raise questions about the most effective ways to engage parents in research given the important role they can play in profiling their child’s language skills.

There is limited research on the language learning profiles of dual language learners, especially dual language learners in areas of significant social disadvantage (83). Our attempt to consider children’s home language use on the same scale was both exploratory and novel. The same unitary structure was evident for the scale in both English and home language. Overall, as expected, children who were monolingual performed better on the language measures in English. However, as the regression analyses indicated, it was not whether children were monolingual that predicted their factor score on the LUI-6 English, but rather their performance in English as assessed by direct measures of oral language. Previous studies have shown better performance on the oral language assessments was associated with more English language exposure and more exposure from native English speakers (8, 84). These are factors which can be addressed in early years settings. This is not to minimize the importance of home language, but rather to empower children in the current language of education in England. In this context, it is noteworthy that overall, where parents reported for English and home language, skills were always reported to be more advanced in English. Further work is required to establish whether these differences reflected the heterogeneity of the population sampled, or the way in which the scale was administered. Performance of language minority pupils is more strongly associated with the concentration of social disadvantage than with the concentration of pupils who speak a different language than the one taught at school [(85) p. 19]. Capturing children’s proficiency across their languages provides opportunities for settings to build on language diversity in an evidence informed way.

### Limitations

Despite our large unique sample and the robust evidence for the validity of the modified version of the LUI, there are several significant limitations which need to be addressed in future studies. Firstly, although many parents completed the form, there was evidence of selective completion of the scale. In particular, even in using the abbreviated LUI, fewer responses were received from parents of bilingual/multilingual children, children with lower language levels, and those from the most deprived settings. The reasons for non-completion are not known, but may reflect engagement with the study, time available to complete the scale, and/or low literacy levels in the parents. This raises concerns both about the representativeness of the data and the importance of supporting completion in these disadvantaged and often multilingual settings. Secondly, our knowledge of children’s home language use was limited. Information about the frequency with which the languages spoken at home was neither available nor did we have data on parents’ English language competence, which limits are understanding of the impact of language status. Finally, although no measures exist to capture all the languages used in the settings we sampled, the LUI-6 was presented in the English language, and this may impact on parental understanding as well as completion rates.

## Conclusions and Future Directions

To close the gaps in the oracy skills of children from lower-SES homes, children need to develop language skills at an accelerated rate (86, 87). Early support in educational settings is critical to address this gap. We reasoned that a tool which supported teachers’ ability to profile children’s language skills would be an important lever to for these early years settings. Parents completed a measure which was simple to score and that provided a profile of children’s language in an efficient way for practitioners. The shortened version has the potential to be used in a range of settings, but may be particularly useful in areas of social disadvantage with more EAL speakers and lower levels of literacy. In sum, the current study demonstrates that the LUI-6 English was an effective measure of language abilities in young children. The data also raise important new avenues for research to capture the language learning needs of children as they enter nursery school. The need to capture economically and linguistically diverse populations in interventions (88) requires the development and use of valid tools. The LUI-6 aimed to capture expressive vocabulary and grammar, and therefore missed other aspects of social pragmatic information. It may be that completion of the 10 main scales of the LUI could capture greater diversity in the population, especially if more reliable and valid data were collected about children’s home language and their use and proficiency in this language. Given the diverse population of children that enter nurseries in areas of social disadvantage, providing teachers with ways of reliably capturing children’s language performance to map progress and evaluate interventions is of paramount importance.

## Data Availability Statement

The raw data supporting the conclusions of this article will be made available by the authors, without undue reservation.

## Ethics Statement

The studies involving human participants were reviewed and approved by the Institute of Education, UCL Ethics Committee. Written informed consent to participate in this study was provided by the participants’ legal guardian/next of kin.

## Author Contributions

JD and JL conceptualized, designed, and managed the study. SM contributed to the work in the early years settings and staff needs. CF identified key subscales for the study and completed all pilot work for the abbreviated measure. CF and JC collected and entered data. All authors except JL were responsible for the final submission. All authors contributed to the article and approved the submitted version.

## Conflict of Interest

The authors declare that the research was conducted in the absence of any commercial or financial relationships that could be construed as a potential conflict of interest.

## Publisher’s Note

All claims expressed in this article are solely those of the authors and do not necessarily represent those of their affiliated organizations, or those of the publisher, the editors and the reviewers. Any product that may be evaluated in this article, or claim that may be made by its manufacturer, is not guaranteed or endorsed by the publisher.

## Footnotes

1 We would like to thank Dr. O’Neill for sharing her measure with us, providing a critical appraisal of our use of the measure and reading the final manuscript.

2 The author of the original LUI is currently working on an abbreviated version of the full measure. Please contact Dr. O’Neill for details.
